# The metabolic costs of improving ethanol yield by reducing glycerol formation capacity under anaerobic conditions in *Saccharomyces cerevisiae*

**DOI:** 10.1186/1475-2859-12-29

**Published:** 2013-03-28

**Authors:** Julien Pagliardini, Georg Hubmann, Sandrine Alfenore, Elke Nevoigt, Carine Bideaux, Stephane E Guillouet

**Affiliations:** 1 Université de Toulouse, INSA, UPS, INP, LISBP, 135 Av. de Rangueil, F-31077 Toulouse, France INRA, UMR792 Ingénierie des Systèmes Biologiques et des Procédés, F-31400 Toulouse, France; CNRS, UMR5504, Toulouse F-31400, France; 2 Laboratory of Molecular Cell Biology, Institute of Botany and Microbiology, Katholieke Universiteit Leuven, Kasteelpark Arenberg 31 - bus 2438, Heverlee, Flanders B-3001, Belgium; 3 Department of Molecular Microbiology, VIB, Kasteelpark Arenberg 31 - bus 2438, Heverlee, Flanders B-3001, Belgium; 4 School of Engineering and Science, Jacobs University gGmbH, Campus Ring 1, Bremen 28759, Germany

## Abstract

**Background:**

Finely regulating the carbon flux through the glycerol pathway by regulating the expression of the rate controlling enzyme, glycerol-3-phosphate dehydrogenase (GPDH), has been a promising approach to redirect carbon from glycerol to ethanol and thereby increasing the ethanol yield in ethanol production. Here, strains engineered in the promoter of *GPD1* and deleted in *GPD2* were used to investigate the possibility of reducing glycerol production of *Saccharomyces cerevisiae* without jeopardising its ability to cope with process stress during ethanol production. For this purpose, the mutant strains TEFmut7 and TEFmut2 with different *GPD1* residual expression were studied in Very High Ethanol Performance (VHEP) fed-batch process under anaerobic conditions.

**Results:**

Both strains showed a drastic reduction of the glycerol yield by 44 and 61% while the ethanol yield improved by 2 and 7% respectively. TEFmut2 strain showing the highest ethanol yield was accompanied by a 28% reduction of the biomass yield. The modulation of the glycerol formation led to profound redox and energetic changes resulting in a reduction of the ATP yield (Y_ATP_) and a modulation of the production of organic acids (acetate, pyruvate and succinate). Those metabolic rearrangements resulted in a loss of ethanol and stress tolerance of the mutants, contrarily to what was previously observed under aerobiosis.

**Conclusions:**

This work demonstrates the potential of fine-tuned pathway engineering, particularly when a compromise has to be found between high product yield on one hand and acceptable growth, productivity and stress resistance on the other hand. Previous study showed that, contrarily to anaerobiosis, the resulting gain in ethanol yield was accompanied with no loss of ethanol tolerance under aerobiosis. Moreover those mutants were still able to produce up to 90 gl^-1^ ethanol in an anaerobic SSF process. Fine tuning metabolic strategy may then open encouraging possibilities for further developing robust strains with improved ethanol yield.

## Background

High yield, final concentration and productivity of the desired product are the major objectives for optimizing microorganisms used in an industrial scale bioprocess. Beyond that, it has to be ensured that the microorganism can still cope with process constraints, which might expose the microorganism to severe stress. Generally speaking, finding an acceptable trade-off between these opposing requirements is a major challenge for successful strain engineering. In particular modifications of the central carbon metabolism are inherently coupled to energy and redox issues [1], which might cause severe side effects on the cell’s robustness towards environmental stress. One prominent example for such a challenge is the reduction of glycerol formation in *Saccharomyces cerevisiae* in order to increase the ethanol yield. Glycerol is one of the main by-products in ethanol fermentation and may account for up to 5% of the substrate carbon [2]. Therefore, the abolishment or at least a substantial reduction may lead to a significant increase in ethanol yield. This issue has been on the scope for a long time and has been addressed by both, process optimisations [3] and genetic engineering [1,4]. Despite of their success in reducing glycerol formation, both approaches often resulted in severe side effects on growth and performance. These studies also substantiated the importance of glycerol as a major player in the cell metabolism [5,6], as a central element of the cell redox balance [7], as essential precursor for phospholipids and triacylglycerolipids [8], and lastly as an essential constituent of the cell stress resistance system [9].

Concerning the role of glycerol in the redox balance, glycerol is involved in the transfer of the reducing power from the cytosol to the mitochondria in aerobic condition, but more importantly it is mainly used as a sink for electrons under anaerobic conditions [7]. Indeed the coupling of glycolysis and ethanol production presents a null oxydo-reductive balance [10], however the synthesis of organic acids as well as some anabolic reactions produce an excess of NADH [10,11]. In anaerobiosis, the glycolytic intermediate dihydroxyacetone phosphate (DHAP) is reduced to glycerol-3-phosphate (G3P) at the expense of one NADH [10] and subsequently, G3P is dephosphorylated into glycerol as the final metabolite [5,12].

Furthermore, glycerol is also known for its broad implication in stress resistance, particularly in osmotic stress. Glycerol is the main compatible solute accumulated in *S. cerevisiae*[13]. Intracellular accumulation of glycerol contributes to maintain turgor pressure and prevents the loss of water under hyperosmotic conditions. Intracellular glycerol concentrations are regulated by the High Osmolarity Glycerol (HOG) MAP kinase pathway [9], which enhances glycerol formation under hyperosmotic stress, and by the plasma membrane channel Fps1, which regulates the efflux rate of glycerol during a hypo-osmotic shock [14]. Apart from osmotic stress, a potential role of glycerol in resistance to a wide range of stress types such as temperature, thawing, oxidative stress as well as stress by high ethanol concentration has been suggested in literature [9]. These broad implications in central cellular functions make it difficult to engineer mutant strains, showing not only the desired reduction of glycerol but also stress robustness.

Approaches aiming at redirecting the main glycerol pathway, mostly targeted the genes encoding for the enzymes directly involved in glycerol formation, namely the glycerol-3-phosphate dehydrogenase (GPDH) and the glycerol-3-phosphatase (GPP). Both enzymes exist in two iso-forms encoded by their corresponding isogenes, which show highly similar sequences. However the physiological role within the cell differs quite considerably among the isoforms [15,16]. The GPDH isoform, Gpd1 is involved in the response to osmotic stress [17] and its activity increases in condition of hyperosmotic stress. Strains with deleted *GPD1* are osmo-sensitive [17]. Gpd2 is involved in the response to anaerobiosis; strains with deleted *GPD2* showed an altered growth under anaerobic conditions and its activity was found increased in absence of oxygen [15]. Mutants being deleted in either one or both of the two isogenes, *GPD1* and *GPD2*, were constructed in different backgrounds [4,12,15,18-20]. Under anaerobic conditions, the *gpd1*Δ and *gpd2*Δ mutant showed an increase in ethanol yield of 2.8% and 4.7% respectively, while the *gpd1*Δ *gpd2*Δ mutant strain was not able to grow. Under aerobic conditions, *gpd1*Δ and *gpd2*Δ increased their ethanol yield by 2.2% and 3.3%, respectively. The *gpd1*Δ *gpd2*Δ mutant increased the ethanol yield by 12.7%, however not only due to abolished glycerol formation but also due to reduction in the biomass yield by 28.8% [21]. Moreover, the assessment of the ethanol production capacity of the *gpd1*Δ *gpd2*Δ mutant in an aerobic high ethanol production showed that its tolerance to ethanol was reduced [21]. Gpp1 and Gpp2 are involved in osmotic stress. However, strains with a deletion of *GPP1* are not able to grow under anaerobiosis. A recent study targeted both GPP iso-enzymes, Gpp1 and Gpp2. This study showed that, in aerobic conditions, deletion of one gene did not affect growth or glycerol production, while deletion of both genes only lead to a 50% decrease in the glycerol formation, suggesting for unspecific glycerol dephosphorylation, or activation and reversion of the catabolic glycerol pathway [21,22].

Alternative strategies to reduce glycerol investigated an altered cofactor use to decrease the need for NADH re-oxidation in the cell, by engineering the redox metabolism. This was either done by i) decreasing the NADH produced or by ii) introducing new reaction consuming NADH. In the first case, an attempt to modify the redox metabolism was made by by-passing the NAD^+^-dependent glycolytic conversion of glyceraldehyde to glycerate through the heterologous expression of a NADP^+^-dependent glyceraldehyde-3-phosphate dehydrogenase. This strategy replaced a NADH producing reaction by a NADPH producing reaction and resulted in a reduction in glycerol yield of 40% and an increase in the ethanol yield by 3%. The biomass yield was not constant throughout the tested strains [23]. One example for new reactions, which consumed NADH and replaced glycerol as redox sink, was carried out by Nissen *et al.*[23]. In this study, the ammonium assimilation was modified by deleting the gene *GDH1* encoding the NADP^+^-dependent glutamate dehydrogenase and over-expressing the genes for the NAD^+^-dependent ammonium assimilation pathway *GLN1*/*GLT1*. This allowed decreasing the need for NADH re-oxidation via glycerol formation and resulted in a reduction in glycerol yield by 38% and an increase of ethanol yield by 10% [23]. In a recent approach, a new pathway for NADH reoxidation was introduced by overexpression of the *Escherichia coli* gene *mhpF*, encoding the acetylating NAD-dependent acetaldehyde dehydrogenase, in a *gpd1*Δ *gpd2*Δ mutant. The reduction of acetate to acetaldehyde in *S. cerevisiae* consumed one NADH instead of the usual NADPH. This reaction provides an alternative redox sink to reoxidize excess NADH. Therefore, it was possible to partly restore growth of the *gpd1*Δ *gpd2*Δ mutant under anaerobic conditions. In this mutant, NADH was completely re-oxidized by the reduction of acetic acid to ethanol via the new NADH-dependent reaction. The co-fermentation of acetic acid together with glucose represents an interesting strain property in ethanol production from lignocellulosic hydrolysates, which contains a significant concentration of acetic acid [24].

Other recent studies combined the modification in the glycerol synthesis pathway, redox metabolism engineering, the modification of yeast glycerol transport systems and the overexpression of trehalose synthesis genes [25-30]. The best results were obtained by deleting the *GPD1*gene, over-expressing the trehalose synthesis genes *TPS1* and *TPS2* and expressing the *Bacillus cereus* glyceraldehyde-3-phosphate dehydrogenase *GAPN*, the strain showing a 75% reduction of glycerol yield concomitant to a 8% ethanol yield increase [30]. Though, those studies were obtained on rich medium (YPD) or without complete product analysis (CO_2_ for example), which did not allow a close monitoring of carbon fate during the fermentation leaving gaps in the understanding of the metabolism in those strains.

Advances in yeast promoters engineering have recently allowed to finely grade gene expression allowing to circumvent a complete gene deletion, which might cause severe side effects [31]. In order to study *S. cerevisiae* strains which have a glycerol formation capacity ranging between that of the *gpd2*Δ single mutant (100%) and the *gpd1*Δ *gpd2*Δ double mutant (0%), we recently replaced the native *GPD1* promoter in a *gpd2*Δ background by two well-characterized *TEF1* promoter mutant versions [31,32]. The genetic modifications were accompanied by 61% and 88% reduction in glycerol yield on glucose and by 20 and 30% reduction in maximal aerobic growth rate compared to the wild type. Interestingly, the engineered (“intermediate”) strains referred to as TEFmut2 and TEFmut7 showed a 2 and 5% increase in ethanol yield and could well cope with process stress, which is in remarkable contrast to a *gpd1Δ gpd2Δ* mutant. These results were obtained in a Very High Ethanol Performance (VHEP) fed-batch process with aeration [32]. Flux calculation based on a metabolic model [3,32,33] showed that the carbon flux through the glycerol pathway was sufficient to provide enough G3P as biomass precursor and to sustain the maximal growth yield, observed in the wild type strain. Under fully aerated conditions, we did not observe a negative impact of low glycerol production upon the industrial relevant traits of the production strain. Results showed that, in such conditions, it was possible to widely decrease the glycerol yield, increase the ethanol yield and limit the negative impact of the deletion in regards to biomass, viability and tolerance to ethanol [32]. Recently, we constructed a collection of different strains with different combinations of residual *GPD1* and *GPD2* expression levels controlled by the TEFmut2 and TEFmut7 engineered promoters [34]. Among our engineered strains we identified four strains showing improved ethanol yields compared to the wild type. In contrast to the *gpd1*Δ *gpd2*Δ mutant, these strains were able to completely ferment the sugars under quasi-anaerobic conditions in both minimal medium and during Simultaneous Saccharification and Fermentation (SSF) of liquefied wheat mash (wheat liquefact) [34]. In the current study, the two strains, TEFmut7 and TEFmut2, were grown in a VHEP fed-batch process under high productivity anaerobic ethanol fermentation. The quantitative kinetic analysis was applied to evaluate the impact of reduced glycerol formation on the overall yeast metabolism and the cell viability.

## Material and methods

### Strains, media and growth conditions

The *Escherichia coli* strain DH5α™ (Invitrogen Corp., Carlsbad) was used for amplification of plasmids. The strain was grown in Luria-Bertani (LB) medium (0.5% yeast extract, 1% peptone, 1% NaCl, pH 7) at 37°C. *E. coli* transformation and isolation of plasmid DNA were carried out using standard techniques [35]. All *Saccharomyces cerevisiae* strains used have been previously described [32,34]. For initial pre-cultivations, yeast strains were grown on YPD plates (2 g L^-1^ glucose, 1 g L^-1^ yeast extract, 1 g L^-1^ bacto peptone, 0.9 g L^-1^ NaCl, 1.5 g L^-1^ agar) and stored in 30% glycerol at −80°C. All yeast strains used in this study are prototrophic allowing the use of minimum mineral media without any amino acid or nucleic base supplementation. All subsequent pre-cultures and fermentation experiments were carried out in synthetic mineral medium prepared as follows (all concentrations in g L^-1^): KH2PO4, 3.0; (NH4)2SO4, 3.0; Na2HPO4 12H2O, 3.0; sodium glutamate, 1.0; MgSO4 7H2O, 0.5; ZnSO4 7H2O, 0.04; MnSO4 H2O, 0.0038; CoCl2 6H2O, 0.0005; CuSO4 5H2O, 0.0009; Na2MoSO4 2H2O, 0.00006; CaCl2 2H2O, 0.023; (NH4)2Fe(SO4)6 6H2O, 0.023; H3BO3, 0.003; pantothenate, 0.005; nicotinic acid, 0.005; meso-inositol, 0.125; thiamine, 0.005; pyridoxine, 0.005; para-aminobenzoic acid: 0.001, and biotin, 0.000012 [36].

Although *S. cerevisiae* can grow under anaerobic conditions in rich medium, there are several biosynthetic pathways such as those for sterols and unsaturated fatty acids that require the presence of molecular oxygen as long as synthetic minimal medium is used. Therefore, sterols and unsaturated fatty acids were added to the medium described above. Sterols were added in form of ergosterol (63 mg L^-1^). Oleate, which can be used as a source for unsaturated fatty acids, was added to the media in the form of TWEEN 80 (2.63 g L^-1^). Both substances were dissolved in pure ethanol. The concentration of ethanol in the ergosterol, TWEEN 80 preparation was calculated and the amount of ethanol, added to the reactor through the supplementation of the preparation, was noted. The added ethanol was later subtracted from the total amount of ethanol measured in the bioreactor.

Three steps of propagation with increasing culture volumes (5 mL, 30 mL, 300 mL) were carried out before inoculating the reactor for the VHEP fed-batch fermentations. Each pre-culture was grown for 12 hours and used as the inoculum for the next step at a 10% v/v ratio.

### Anaerobic fed-batch protocol

Anaerobic fed-batch fermentations were carried out in 5 L bioreactors B DCU B.BRAUN (SARTORIUS) with a starting volume of 3 L. Temperature was set at 30°C and pH regulated at 4 by adding 14% (v/v) NH_3_ solution. Reactors were flushed with a constant 0.5 l min^-1^ N2 flux throughout the experiment. Aeration started not before 1 hour after the inoculation in order to accumulate CO_2_ in the medium and prevent growth limitation due to CO_2_ stripping phenomenon. A sequential vitamin feeding strategy based on the growth profile [36] was applied. The fermentations were started with an initial glucose concentration of 100 g L^-1^. Whenever the residual glucose concentration was lower than 20 g L^-1^, glucose was fed to restore a glucose concentration of 100 g L^-1^. The glucose fed was adjusted to 50 g L^-1^ at the later phase of the fermentation when growth had stopped.

### Gas analysis

Outlet gas analysis was performed on the outlet flow of the reactor using a mass spectrometer Proline Dycor^2^a (Ametek Process Instrument) every 5 minutes. The CO_2_ production rate was calculated from the differences between the inlet and outlet gas compositions, taking into account the evolution of the liquid volume in the reactor, the inlet airflow (regulated by a mass flowmeter), the temperature and the pressure.

### Analytical methods

Yeast growth was evaluated by spectrophotometric measurements at 620 nm in a spectrophotometer Libra S4 (Biochrom) and calibrated against cell dry weight measurements. Cells were harvested by filtration on 0.45-μm-pore-size polyamide membranes (Sartorius Biolab Product) and dried to a constant weight at 60°C under a partial vacuum (200 mm Hg ~ 26.7 kPa) for 24 hours. Rapid determination of glucose and ethanol concentrations from broth supernatants during fermentation was performed with an YSI analyser (YSI model 27 A; Yellow Springs Instruments).

Determination of ethanol, organic acids and glucose from supernatants was performed by HPLC using an Aminex HPX-87H+ column (300 mm ×7.8 mm) and dual detection (refractometer and UV at 210 nm) at 50°C with 5 mM H2SO4 as an eluant (flow rate of 0.5 L min–1). Technical triplicates (taking into account sampling, separation and HPLC quantification) showed that the measurement was reproducible; typically standard deviation was lower than 1% of mean value for ethanol and glucose and lower than 5% for glycerol and acetate.

### Determination of the cells' viability

To determine cells' viability, the methylene blue technique was used as previously described [36].

### Assessment of ethanol evaporation

The part of evaporated ethanol was taken into account in the mass balances as previously described [32].

### Metabolic flux calculations

Metabolic fluxes were calculated using a MFA based model extrapolated from the previously version described in [32] in order to account specific physiology under anaerobic conditions according to literature and previously described models [37-42]. The oxidative phosphorylation reactions were suppressed. The lanosterol production reaction from Acetyl-CoA was also suppressed and ergosterol was added as a substrate for the other sterols production. In the Krebs cycle the succinyl-CoA synthase catalysed reaction was removed. An ethanol shuttle for the redox equivalent translocation between cytosol and mitochondria was added. The mitochondrial NADPH was assumed to come from the NADP^+^ dependant isocitrate deshydrogenase under anaerobiosis and a NADH dependant glutamate synthesis reaction was added in the cytosol. Our final metabolic network consisted in 129 reactions, including 14 exchange reactions between the cell and external medium, 64 cytosolic reactions, 27 transport reactions between mitochondria and cytosol, and 14 mitochondrial reactions (cf. Additional file 1). Validity of the model was assessed using published data based on ^13^C labelling experiments [43,44].

The Y_ATP,X_ values and NADH balance were calculated from the results of metabolic flux calculation as follows:

$$Y_{\mathrm{ATP},X}=\frac{\mu }{\sum_{i}\alpha_{i}^{\mathrm{ATP}}\times q_{i}^{\mathrm{Glycolysis}}}$$

$$\begin{array}{l} \mathrm{NAD}H_{\mathrm{produced}}=\sum_{i}\alpha_{i}^{\mathrm{NADH}}\times q_{i}^{\mathrm{Glycolysis}}+\sum_{i}\alpha_{i}^{\mathrm{NADH}}\\ \;\times q_{i}^{\mathrm{TCA}}+\sum_{i}\alpha_{i}^{\mathrm{NADH}}\times q_{i}^{\mathrm{Anabolism}} \end{array}$$

$$\begin{array}{l} \mathrm{NAD}H_{\mathrm{consummed}}=\alpha_{\mathrm{Succinate}}^{\mathrm{NADH}}\times q_{\mathrm{Succinate}}+\alpha_{\mathrm{Glycerol}}^{\mathrm{NADH}}\\ \;\times q_{\mathrm{Glycerol}} \end{array}$$

$\alpha_{i}^{x}$: Stoichiometric coefficient of metabolite x in reaction i

$q_{i}^{y}$; Rate of reaction i belonging to metabolic pathway y

The uncertainties of the TCA cycle fluxes values were estimated to impact the Y_ATP,X_ and NADH balance by less than 5%.

## Results

### Impact of the reduced GPDH activity on fermentation parameters

The two engineered strains, TEFmut7 and TEFmut2, exhibiting a residual GPDH activity of 55% (0.023 U/mg protein) and 6% (0.006 U/mg protein) when compared to wild type activity, were cultivated in an anaerobic fed-batch fermentation in a synthetic mineral medium.

**Figure 1:**
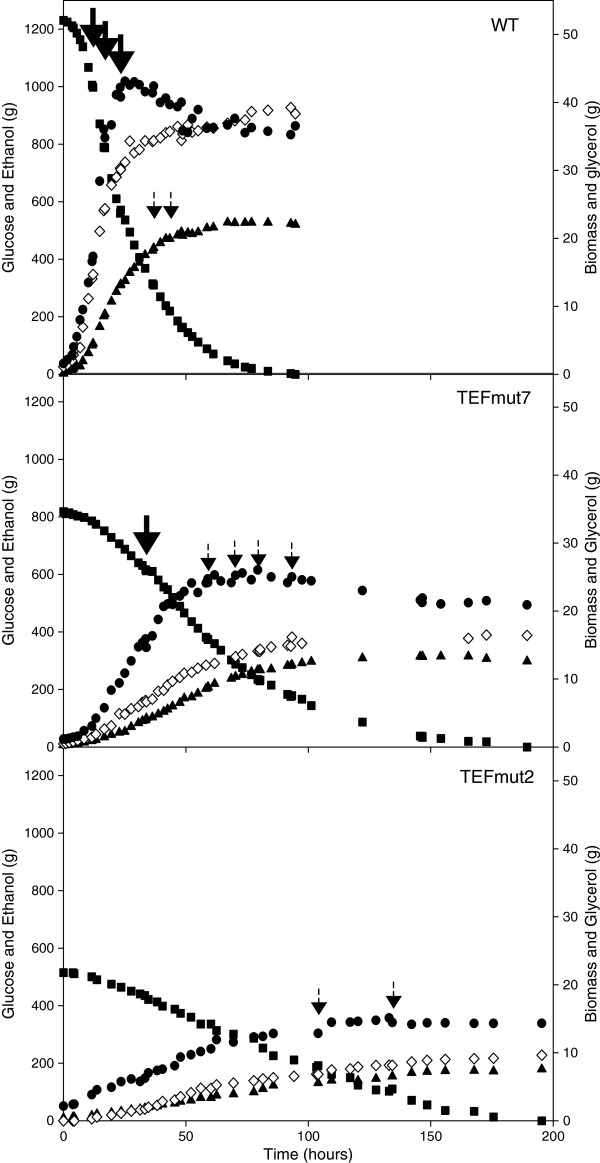
**Time-courses of the mass of glucose, ethanol, biomass and glycerol in the fed-batch cultivations.** Strains: Wild Type CEN.PK113-7D (WT), TEFmut7, and TEFmut2 strains. Mass of glucose (■), ethanol (▲), biomass (●) and glycerol (◇). Thick arrows indicate the time points when glucose feeding was carried out in order to adjust glucose concentration in the bioreactor up to 100 g L^-1^, thin arrows correspond to glucose feeding up to a concentration of 50 g L^-1^.

Time courses of glucose consumption, biomass, ethanol and glycerol production of the CEN.PK 113-7D, TEFmut7 and TEFmut2 are shown in Figure 1. All three fermentations showed two characteristic phases: a first “growth phase” where biomass was produced concomitantly with ethanol, and a second “uncoupled production phase”, where growth had stopped due to ethanol inhibition but cells kept on producing ethanol. Growth of the wild type ended after about 24 hours; growth of the mutants TEFmut7 and TEFmut2 ended after 60 and 133 hours, respectively. Ethanol production stopped after about 70 hours for the wild type, 146 hours and 167 hours for TEFmut7 and TEFmut2, respectively. Noteworthy, the uncoupled ethanol production phase was very short in the case of the mutant TEFmut2. Production phase represented about 66% of the overall fermentation time for wt, 59% for TEFmut7 and only 20% for TEFmut2. Hence 61% of the total ethanol was produced during the growth for the wt, 59% for TEFmut7 and 89% in the case of TEFmut2.

Calculated growth rate, biomass yield, metabolite yields, final titer and productivity are summarized in Table 1. Carbon balances were closed to 95% for the wild type, to 88% for TEFmut7 and to 87% for TEFmut2; the degree of reduction balances closed to 83%, 85% and 80%, respectively. Evaluation of evaporated ethanol resulted in carbon and degree of reduction balances above 95% for all three fermentations.

**Table 1:** **Fermentation characteristics for *Saccharomyces cerevisiae *CEN.PK113-7D and the two mutant strains during anaerobic fed-batch fermentation at 30°C**

	**WT**	**TEFmut7**	**TEFmut2**
Fermentation time (h)	70	146	167
Growth time (h)	24	60	133
μmax (h^-1^)	0.29 ± 0.01	0.13 ± 0.01	0.046 ± 0.001
DCWmax (g L^-1^)	12.0 ± 0.5	8.0 ± 0.5	3.8 ± 0.2
Y DCW/glucose g g^-1^	0.076 ± 0.001	0.074 ± 0.003	0.055 ± 0.001
	[0–16 h]	[0–30 h]	[0–44 h]
Y glycerol/glucose g g^-1^	0.056 ± 0.001	0.031 ± 0.003	0.022 ± 0.004
	[0–16 h]	[0–30 h]	[0–44 h]
Y glycerol/DCW g g^-1^	0.74 ± 0.03	0.42 ± 0.02	0.40 ± 0.04
	[0–16 h]	[0–30 h]	[0–44 h]
Y acetate/ glucose g g^-1^	0.005 ± 0.001	n.d.	n.d.
Y pyruvate/ glucose g g^-1^	0.003 ± 0.001	n.d.	n.d.
Y succinate/ glucose g g^-1^	0.004 ± 0.001	0.013 ± 0.001	0.026 ± 0.001
Yethanol/glucose g g^-1^ (growth)	0.45 ± 0.003	0.46 ± 0.008	0.48 ± 0.005
Yethanol/glucose g g^-1^ (overall)	0.47 ± 0.001	0.48 ± 0.003	0.48 ± 0.005
[ethanol] final (g L^-1^)	139 ± 1	106.0 ± 0.5	57 ± 2
[glycerol] final (g L^-1^)	9.0 ± 0.3	5.4 ± 0.1	2.8 ± 0.2
Ethanol productivity (g L^-1^ h^-1^)	1.97 ± 0.10	0.73 ± 0.03	0.33 ± 0.02
Pcritical (g L^-1^)	90 ± 2	78 ± 1	54 ± 2

μ_max_: maximum specific growth rate, DCW_max_ maximum cell concentration , Y_i/j_ yield of production of constituent *i* on the constituent *j*: DCW biomass ;.n.d. : not detected (detection thresholds were 60, 50 and 100 mg L^-1^ for acetate, pyruvate and succinate respectively). Yields, concentrations, and specific rates are average values of 2 independent fermentations. Productivity and fermentation time correspond to the experiments shown in the paper.

The maximum biomass concentration established at 12 g L^-1^ for the wt, 8 g L^-1^ for TEFmut7 and 3.8 g L^-1^ for TEFmut2. Final glycerol concentration established at 9 gL^-1^ for the wt strain compared to 5.4 g L^-1^ for TEFmut7 and 2.8 g L^-1^ for TEFmut2. Final ethanol concentration reached was reduced to 106 g L^-1^ for TEFmut7 and 57 g L^-1^ for TEFmut2 compared to 139 g L^-1^ for the wild type (Table 1).

### Reduced glycerol formation altered fermentation rates

**Figure 2:**
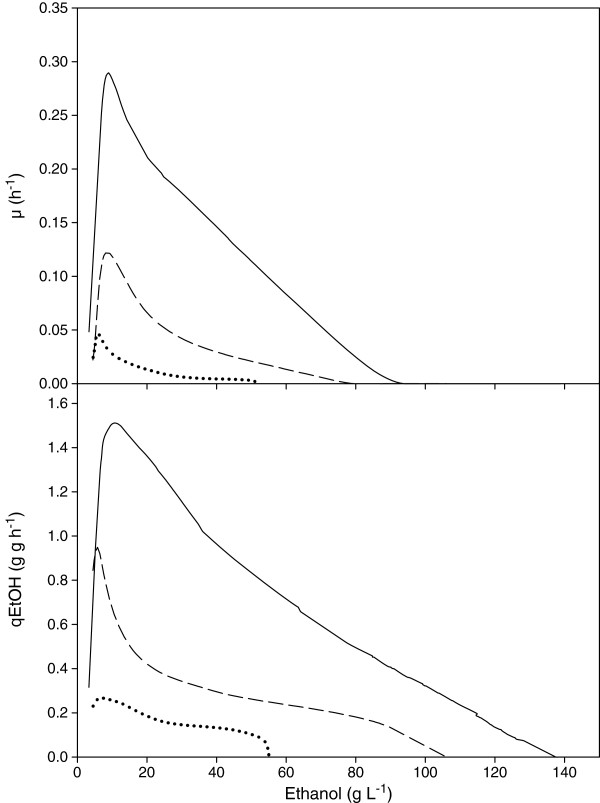
**Specific growth rate (μ) and specific ethanol production rate (qEtOH) as functions of ethanol concentration.** Strains: wild type CENPK113-7D ( ___ ), TEFmut7 ( − − - ), TEFmut2 (......).

The reduction of GPDH activity in the engineered strains led to a decrease in the maximum specific glycerol production rate of 64 and 92%, respectively in TEFmut7 and TEFmut2 compared to the wild type (0.2 g glycerol g DCW^-1^ h^-1^) (Figure 2). However, the maximum specific growth rate and the maximum specific ethanol production rates were also reduced in TEFmut7 and TEFmut2. The maximum growth rate, μ_max_ was 0.29 h^-1^ for the wild type, 0.13 h^-1^ for TEFmut7, and 0.046 h^-1^ for TEFmut2. The maximum specific ethanol production rates were reduced by 57% and 85% in TEFmut7 and TEFmut2, respectively compared to the wild type (1.5 g ethanol g DCW^-1^ h^-1^). The overall volumetric ethanol productivity was also affected in TEFmut7 and TEFmut2, i.e. 0.73 and 0.33 g L^-1^h^-1^ respectively, compared to 1.97 g L^-1^h^-1^ for the wild type (Table 1).

### Reduction in the glycerol formation improved the ethanol production yield

The reduced GPDH activity in the two engineered strains led to a reduction of the glycerol yield on glucose by 44% for TEFmut7 and 61% in TEFmut2 compared to the wt strain. The glycerol production yield per g of biomass was equal to 0.74 g g DCW^-1^ for the wild type and 0.42 g g DCW^-1^for TEFmut7. Surprisingly, no relevant difference in the glycerol yield per g of biomass was observed between TEFmut7 and TEFmut2 (0.40 g g^-1^_DCW_). The overall ethanol yield on glucose established at 0.48 g g^-1^ for TEFmut7 and TEFmut2 compared to 0.47 g g^-1^ for the wt strain. Differences in the ethanol yields were only observed during the growth phase resulting in yields of 0.45 g g^-1^ for the wt, 0.46 g g^-1^ for TEFmut7 and 0.48 g g^-1^ for TEFmut2. After the growth stopped no significant variation of the ethanol yield could be observed between the three strains. Concomitantly, the biomass production yield on glucose in TEFmut7 and TEFmut2 was 0.074 g_DCW_ g_glucose_^-1^ and 0.055 g_DCW_ g_glucose_^-1^ compared to 0.076 g_DCW_ g_glucose_^-1^ for the wt. Acetate and pyruvate yield were reduced in the mutants and succinate yield was increased (cf. Table 1).

### Comparative analysis of ethanol tolerance of the strains

**Figure 3:**
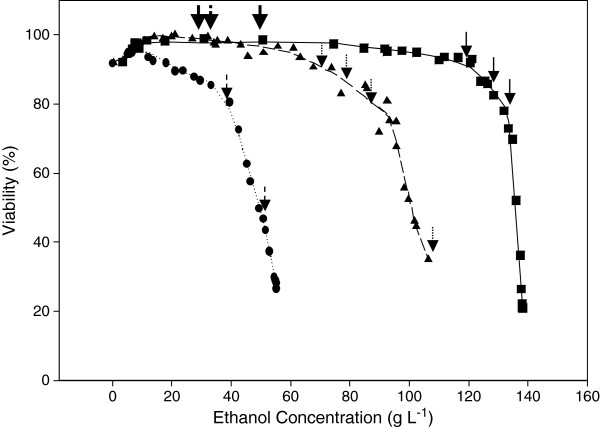
**Cell viability as a function of Ethanol concentration.** Strains: wild type CENPK113-7D (■), TEFmut7 (▲), TEFmut2 (●). Thick Arrows correspond to 100 g L^-1^ glucose addition, thin arrows correspond to 50 g L^-1^ glucose addition. Full line arrows correspond to wild type CENPK113-7D, dut arrow corresponds to TEFmut7 fermentation, and dash arrow to TEFmut2 fermentation.

Figure 2 shows the evolution of both the specific growth and ethanol production rates as a function of the actual ethanol concentration in the bioreactor for each strain. The ethanol concentration at which uncoupling between growth and ethanol production occurs is referred to as P_critical/μ_. This parameter characterizes the strain-dependent growth inhibition by ethanol. P_critical/μ_ was about 90 g L^-1^ for the wild type, 78 g L^-1^ and 54 g L^-1^ for TEFmut7 and TEFmut2, respectively. The cell viability of the mutants in the presence of ethanol was harshly reduced compared to the wild type (Figure 3). Loss of viability profiles showed 3 different dynamics for each of the strains. A first phase was observed during which the strains maintained a constant viability. This first phase lasted for the TEFmut2 until an ethanol concentration of 20 g L^-1^ was reached. In TEFmut7 and the wild type strain, this phase lasted until the ethanol concentration reached 65 and 90 g L^-1^ respectively. The viability decreased slightly in a second phase until an ethanol concentration of 35, 90 and 130 g L^-1^ for the TEFmut2, TEFmut7 and wild type strain, respectively. Further increasing ethanol concentration caused a rapid drop in the cell viability of all three strains.

### Comparative metabolic flux analysis of the strains

**Figure 4:**
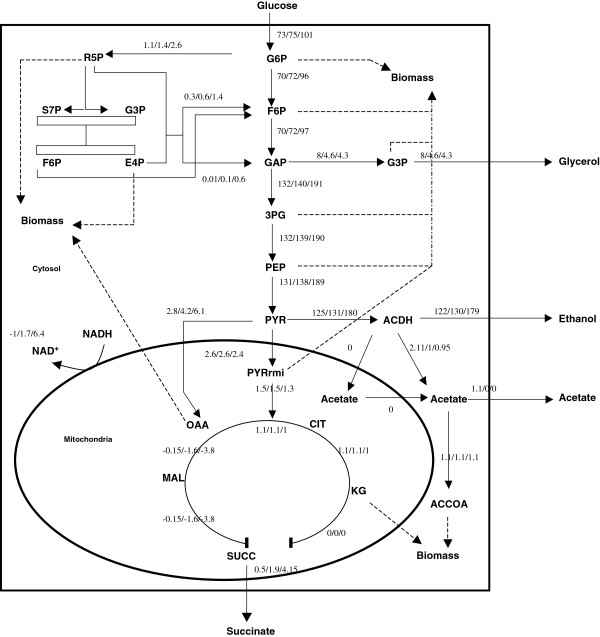
**Metabolic flux repartition within the central carbon metabolism for the 3 *S. cerevisiae *CEN.PK 113-7D strains.** Flux results for wild type/ TEFmut7/ TEFmut2. Metabolic fluxes were calculated as mmol gDCW^-1^ h^-1^ from experimental data obtained at μmax. Fluxes were then normalized to a biomass production rate of 1 g g^-1^ h^-1^. NADH/NAD arrow on the mitochondrion membrane represented the flux of mitochondrial shuttles for reducing power translocation.

Metabolic flux calculations were carried out for each strain to follow up the changes in the metabolism and the metabolic reorganization caused by the modulation of the glycerol synthesis. However, in order to take into account the differences in μmax between the strains, the experimentally obtained specific consumption and production rates were chosen at μ_max_ and normalized to a biomass production rate of 1 g g^-1^ h^-1^. The results of this calculation are reported in Figure 4. It showed that the DHAP-to-G3P flux at μ_max_ was 80% and 53% in TEFmut7 and TEFmut2, respectively compared to the level observed in the wild type. Moreover flux calculation also indicated that the modulation of the glycerol pathway led to a global metabolic reorganization pointed out by the increased normalized rates in ethanol production, glycolysis, NADH mitochondrial shuttles and succinate production.

## Discussion

In order to determine to what extent glycerol formation may be reduced without drastically affecting the strain’s robustness in ethanol production processes, glycerol formation capacity of *S. cerevisiae* was modulated by replacing the native *GPD1* promoter in the *gpd2*Δ background of CEN.PK113-7D with two promoters of significantly lower activities (TEFmut7 and TEFmut2; [31]). For simplicity, the two resulting strains have been referred here to as TEFmut7 and TEFmut2. We previously showed that this glycerol fine-tuning strategy allowed increasing the ethanol yield under both aerobic and anaerobic conditions [32,34]. Under aerobic conditions, the mutant strains could well cope with process stress which is in remarkable contrast to a *gpd1*Δ *gpd2*Δ double deletion mutant. In contrast to the *gpd1*Δ *gpd2*Δ mutant, TEFmut7 and TEFmut2 were able to grow and ferment under anaerobic conditions.

The current work investigated the impact of reduced GPDH activities on the metabolism and strain robustness in an anaerobic VHEP fed-batch process in order to quantify (i) the resulting metabolic changes, particularly the redirection of the carbon flux from glycerol to ethanol and to evaluate (ii) cell viability in the presence of high ethanol concentrations.

### Fine-tuning of the glycerol synthesis pathway led to increased ethanol yield and profound metabolic changes

**Figure 5:**
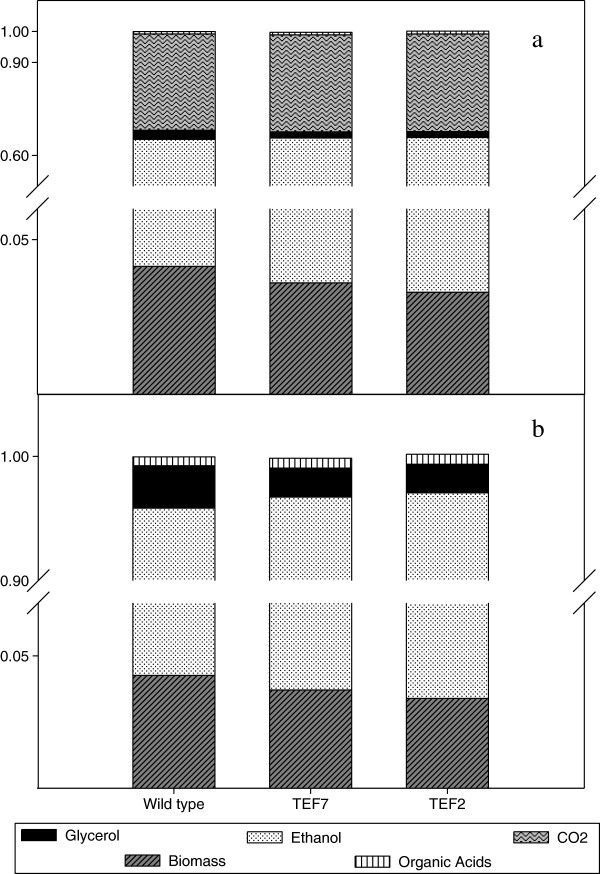
**Normalized carbon (a) and degree of reduction (b) balances during the fermentation of the wild type strain and the two mutants TEFmut7 and TEFmut2.** Data are expressed as Cmol ratio between the amount of metabolites produced (based on final masses at the end of fermentation) and glucose consumed.

**Figure 6:**
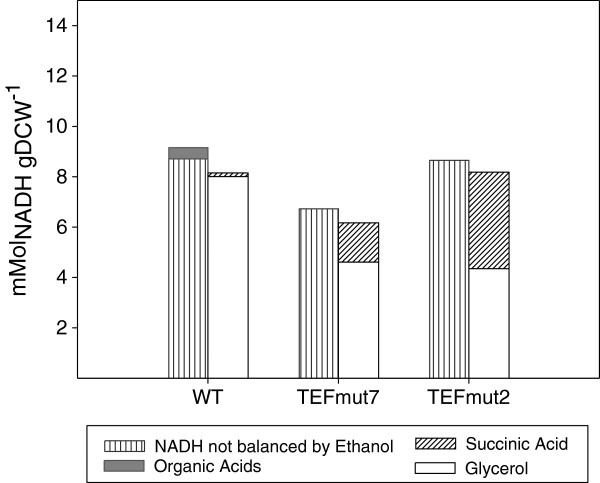
**NADH balances for the *S. cerevisiae *wild-type and the two mutants TEFmut7 and TEFmut2.** NADH balances were calculated for each strain when μmax was reached, respectively from the metabolic flux calculation. Left bars correspond to the generated NADH not balanced by ethanol formation and by pyruvate formation; right bars correspond to the reoxidized NADH (by glycerol and succinate formation).

TEFmut7 and TEFmut2 showed a 44 and 61% reduction of the glycerol yield compared to the wild type. As expected, the reduced glycerol yield was accompanied by an improvement in ethanol yield. During the first phase of the fermentation, the increase in ethanol yield was 2% for TEFmut7 and 7% for TEFmut2*,* which was significant with regard to the respective standard deviations*.* This improvement in ethanol yield was not only due to the reduction of glycerol yield but also resulted from a decrease in the biomass yield and the changes in organic acid formation (Figure 5). The decrease in the biomass yield was already observed under aerobic conditions for these strains [32]. However the anaerobic conditions resulted in a decrease in acetate and pyruvate yield and an increase in succinate yield when compared to the aerobic conditions. The formation of pyruvate from 1 mole glucose generates 1 mole of reduced redox equivalents respectively. In contrast, the synthesis of succinate, which is produced via the reductive branch of the TCA cycle under anaerobic conditions, requires a net reoxidation of 1 mole of NADH. Taking the redox balance into account (Figure 6), the increase in the succinate formation might account for the reoxidation of 20% and 40% of the NADH excess linked to the glycerol reduction in TEFmut7 and TEFmut2, respectively. Taken together with the reduction in the formation of acetate and pyruvate, these modifications were sufficient to counterbalance the decrease in the glycerol yield. Such a metabolic reorganisation in the mutant strains compared to the wild type might be caused by an increase in the NADH/NAD^+^ ratio. This higher NADH/NAD^+^ ratio would hamper the NAD^+^ reducing reactions (acetate, growth), and on the contrary favour the reactions consuming the redox power in the cell (succinate). Noteworthy, the acetate brought in the medium at the beginning of the growth by the inoculum was consumed by the mutant strains (data not shown). Acetate consumption would be an additional way to decrease a high NADH/NAD^+^ ratio [24].

### Reduced glycerol formation altered growth rate, biomass yield and viability in anaerobic fermentation

The total abolishment of glycerol formation is known to completely suppress growth of *S. cerevisiae* under anaerobic conditions [4,12]. In contrast, the reduced but not abolished GPDH activity in TEFmut7 and TEFmut2 allowed the strains to redirect carbon fluxes in a way that the cell could solve the redox-balance issue under anaerobic conditions and provided growth. Unfortunately, the decreased GPDH activity did not only lead to a carbon shift from glycerol towards ethanol but also led to severe side effects on the growth rate and on the cell viability. More precisely, the maximum specific growth rate was reduced by 55% for TEFmut7 and by 84% for TEFmut2 (cf. Figure 2). Despite its low maximum growth rate, the biomass yield of TEFmut7 was not significantly affected by the reduction in GPDH activity. In contrast, TEFmut2 showed a biomass yield reduced by 28%, compared to the wild type during the growth phase (0.055 g_DCW_/g_glucose_ versus 0.076 g_DCW_/g_glucose_). Cell viability and stress tolerance towards ethanol and hyperosmotic shock were considerably affected in TEFmut7 and TEFmut2. Especially the ethanol tolerance was significantly lower in the mutant strain evidenced by a lower P_critical/μ_, lower final ethanol concentration and an earlier viability drop compared to the wild type (Figure 3).

Interestingly, the glycerol yield on biomass yield obtained for TEFmut7 and TEFmut2 was similar despite the fact that the promoter used to control *GPD1* activity in TEFmut7 was more than two-fold stronger than the one in TEFmut2. Both mutants TEFmut7 and TEFmut2 showed a reduction of 43 and 46% in the glycerol/biomass yield. This may suggest that the glycerol/biomass yield observed in the two strains corresponds to the minimum required glycerol formation necessary to maintain cells’ growth and viability.

### Reduced glycerol formation disturbed energetic metabolism in the cell

Under aerobic conditions, the reduction of the glycerol formation increased the energy necessary for the growth resulting in a decreased ATP/Biomass yield (Y_ATP_) [32]. This was attributed to higher energy requirements for the active transport systems involved in the control of the cell homeostasis. A similar phenomenon was observed under anaerobic conditions (cf. Figure 4). The Y_ATP_ of the TEFmut2 mutant calculated by the metabolic model (2.8 g_DCW_.mol_ATP_^-1^) was lower to the one of the wild type (3.8 g_DCW_.mol_ATP_^-1^) and the TEFmut7 mutant (3.6 g_DCW_.mol_ATP_^-1^). Thus, the costs of homeostasis-related transport processes may have even increased compared to aerobic conditions.

The changed ATP energy balance in TEFmut7 and TEFmut2 led to a decrease in the biomass yield but might also caused the mutants' reduced ethanol tolerance. It is commonly assumed that increasing ethanol concentration increases the energy required for growth but also decreases the ability of cells to produce energy by an inhibitory effect on glycolytic enzymes [45]. The maximum energy production rate was 66 mmol ATP.g_DCW_^-1^.h^-1^ for the wild type and 41 and 12 mmol ATP.g_DCW_^-1^.h^-1^ for TEFmut7 and TEFmut2, respectively. A reduced glycerol formation and the accompanied reduction in ATP formation possibly increased the negative effect of ethanol.

Beside the metabolic rearrangements in the cells, a shift in the NADH/NAD^+^ ratio might also cause the reduced capacity to produce energy. An increased NADH/NAD^+^ ratio is known to negatively impact glycolysis rates and thus energy production of micro-organism under anaerobic conditions [45,46]. This reduced energy production rate together with the increased energy requirement may be responsible for the reduced tolerance to ethanol.

### Aeration regime strongly affected the fermentation performances of the mutants with fine tuned glycerol formation

Although fine tuning the glycerol formation pathway improved the ethanol production yield in both aerobic and anaerobic conditions, it significantly lowered the ethanol tolerance of the mutants particularly in anaerobic conditions leading to reduced growth and ethanol production [32]. The decrease in the Y_ATP_ observed in the mutant strains could not completely explain the difference of tolerance between aerobiosis and anaerobiosis since it was within a similar range of magnitude; 5 and 20-25% for TEFmut7 and TEFmut2 respectively.

It is well known that organic acids can affect yeast viability. Comparing the two aeration regimes, the total amount of organic acids per biomass produced during the growth of the mutant strains was within the same range. TEFmut7 and TEFmut2 produced [2-4] and [2.5-3] mmol/gDCW, respectively, independently of the aeration mode. However the nature of the organic acids produced was strongly modified in the mutant strains between the two aeration regimes. Anaerobic conditions were characterized by a higher production of succinate and no production of acetate and pyruvate in the *TEFmut* strains. Possibly the nature of the organic acids produced could explain the reduction in the ethanol tolerance under anaerobic conditions if the intracellular accumulation factors (i.e. ratio between the intra and extra-cellular acid concentrations) are taken into account. These accumulation factors depend on the nature of the acid (notably the pKa values) as described by the Henderson-Hasselbach equation. Following this equation, the accumulation factor for succinate is indeed expected to be more than 10-times higher than the ones for acetate and pyruvate. Therefore the total intracellular acidity could be much higher in the mutants under anaerobic conditions than in the wild type. However it is difficult at this stage to determine to what extent the intracellular acidity may be different between the strains because the potential presence of active exporter for organic acids cannot be overlooked. Piper et al. [47] evidenced the presence of such exporter for acetic acid in *Saccharomyces cerevisiae*. To our knowledge exporter for succinic acid has not been identified so far.

## Conclusions

Mutant strains fine-tuned for their glycerol synthesis capacities showed decreased glycerol yield and improved ethanol yield compared to the wild type strain in anaerobic fermentation. However, contrarily to what was previously observed in aerobic VHEP fermentations, the reduction of the glycerol yield also led to severe reduction in the fermentation kinetics, in cell viability, and in the final ethanol concentration reached. However those mutant strains were able to produce up to 90 g L^-1^ ethanol in an SSF process [34] pointing out that the stress dynamics encountered during the process were important on the fermentation performances linked to yeast viability. Those mutants may then open new routes for metabolic engineering approaches, which provide alternative pathways for NADH reoxidation. Lately, Guo *et al.* succeeded to engineer a yeast strain with lower glycerol formation without influencing its fermentation performance [30]. Those different approaches give encouraging possibilities for further developing robust strains with improved ethanol yield.

## Competing interests

The authors declare that they have no competing interests.

## Authors' contributions

JP, SG and CB contributed to the metabolic model set-up and flux calculation. GH carried out the genetic work. GH, JP, SG, and CB contributed to the fermentations experiments. SG, EN, CB, and SA conceived the study, and participated in its design and coordination and helped to draft the manuscript. All authors read and approved the final manuscript.

## Supplementary Material

Additional file 1 Complementary file. Click here for file
